# Screening and Identification of Novel Ochratoxin A-Producing Fungi from Grapes

**DOI:** 10.3390/toxins8110333

**Published:** 2016-11-12

**Authors:** Xiaoyun Zhang, Yulin Li, Haiying Wang, Xiangyu Gu, Xiangfeng Zheng, Yun Wang, Junwei Diao, Yaping Peng, Hongyin Zhang

**Affiliations:** 1 School of Food and Biological Engineering, Jiangsu University, 301 Xuefu Road, Zhenjiang 212013, China; zhangxiaoyungu@126.com (X.Z.); wanghaiying914@163.com (H.W.); 18796002921@163.com (X.Z.); 13952967706@163.com (Y.W.); wenjun414727@163.com (J.D.); 15751011009@163.com (Y.P.); 2 Hubei Key Laboratory, Edible Wild Plants Conservation and Utilization, 11 Cihu Road, Huangshi 435002, China; liyulin7226@163.com; 3 School of Grain Science and Technology, Jiangsu University of Science and Technology, 2 Mengxi Road, Zhenjiang 212003, China; sdxyugu@126.com

**Keywords:** screening, identification, ochratoxin A (OTA), fungi, grapes

## Abstract

Ochratoxin A (OTA) contamination has been established as a world-wide problem. In this study, the strains with the ability of OTA production were screened by analyzing the green fluorescence of the isolates colonies from the grapes in Zhenjiang with 365 nm UV light and confirmed by HPLC with fluorescent detection (HPLC-FLD). The results showed that seven isolates acquired the characteristic of the fluorescence, of which only five showed the ability of OTA production as confirmed by HPLC-FLD analysis. The five OTA-producing strains were identified based on comparative sequence analysis of three conserved genes (*ITS*, *BenA* and *RPB2*) of the strains, and they are *Talaromyces rugulosus* (O1 and Q3), *Penicillium commune* (V5-1)*, Penicillium rubens* (MQ-5) and *Aspergillus aculeatus* (MB1-1). There are two *Penicillium* species of the five OTA-producing strains and our study is the first to report that *P. rubens*, *T. rugulosus* and *A. aculeatus* can produce OTA. This work would contribute to comprehensively understanding the fungi with an OTA-producing ability in grapes before harvest and then take effective measures to prevent OTA production.

## 1. Introduction

Many fungi produce mycotoxins that can cause acute or chronic intoxication and damage to humans and animals after ingestion of contaminated food and feed [1]. Among the mycotoxins, aflatoxins and ochratoxin A (OTA) occupy special places due to their high occurrence, widespread distribution and acute toxicity. As one of the first group of secondary metabolites of fungi that are toxic to animals, OTA, like aflatoxins started the distinctive science of mycotoxicology in the 1960s [2,3]. OTA is the most toxic member of the Ochratoxin group that includes OTA, its methyl ester, its ethyl ester (OTC), 4-hydroxyochratoxin A (4-OH OTA), ochratoxin B (OTB) and its methyl and ethyl esters and ochratoxin α (OTα). OTA is nephrotoxic, hepatotoxic, teratogenic and immunotoxic to animals and has been associated with fatal endemic human nephropathies [4,5]. Toxicity studies have revealed that OTA mainly affects the kidney and liver, after absorption from the gut and circulation via the portal vein, inducing hepatotoxicity in rats and hepatocellular carcinomas in mice [6]. The European Commission has established maximum allowable limits for this toxin in some food products. A tolerable daily intake (TDI) of no more than 5 ng OTA/kg bw/day is recommended [7].

The occurrence of OTA was mainly attributed to contamination by toxigenic molds with the ability of toxin production before or after harvesting. OTA is a ubiquitous mycotoxin produced by *Aspergillus* and *Penicillium* species that can be detected in a wide variety of agricultural commodities, livestock products and processed food [7]. OTA was firstly described as a secondary metabolite of *Aspergillus ochraceus* from laboratory experiments [2]. In addition, there have been reports about the production of OTA by other strains, mainly *Aspergillus carbonarius* and a small percentage of isolates of the closely related species *Aspergillus niger* [8,9,10,11,12,13,14]*.* Soon after the isolation of OTA from *A. ochraceus*, the formation of OTA by *Penicillium viridicatum* was reported [3]. The species was later correctly identified as *Penicillium verrucosum* [15,16,17]. In fact, the natural occurrence and practical importance of OTA was firstly linked to *Penicillium* species [18,19]. Afterwards, some other OTA-producing strains of *Penicillium* genus were isolated and all the OTA-producing strains were separated into two large groups: *P. verrucosum* and *Penicillium nordicum* [17,20]. In summary, *A. ochraceus* and *P. verrucosum* were the main OTA producing species. However, *A. carbonarius*, as another major source of OTA, has also been identified mainly in grapes [21].

Although cereals are known to be particularly susceptible to OTA contamination [22,23], there is an increasing occurrence of OTA in a large variety of foods, such as beer [24,25] and milk [26]. OTA has also been recently reported in grapes and grape juices [27,28,29,30]. The occurrence of OTA in wine is linked to the presence of molds on grapes. Previous studies have shown that the occurrence of OTA is likely due to environmental conditions (climate) and badly controlled harvesting procedures, poor drying and inadequate storage conditions [31,32].

The objectives of this study were to screen the natural OTA-producing fungi related to preharvest decay and OTA contamination of grapes and test their OTA production capacity in vitro with special attention to those not reported in literature.

## 2. Results

### 2.1. Preliminary Screening of OTA-Producing Fungi by 365 nm Ultraviolet Light

**Figure 1:**
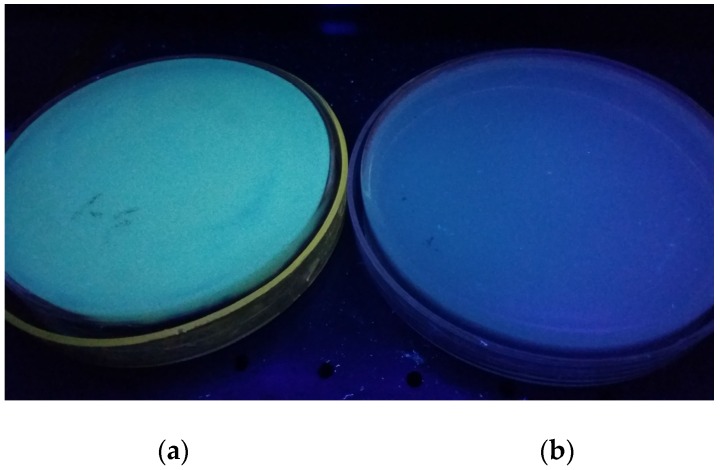
Green fluorescence (**a**) on the reverse of the colony of O1 (an Ochratoxin A (OTA)-producing strain) grown on Czapek’s Agar medium (CA) when was exposed to long wavelength UV light (365 nm). No fluorescence (**b**) was displayed by a non-OTA-producing strain in the same conditions.

One hundred and thirty-nine molds were isolated by macroscopic characters (colony growth and colony morphology) and microscopic characters. The isolates were cultured for 7 or 14 days to accumulate metabolites in the solid culture medium. In 365 nm ultraviolet light, there was one suspected *Aspergillus* isolate and six other isolates that produced green fluorescence in culture medium. After fumigating with 25%–28% ammonia, all seven of the isolates took on enhanced fluorescence intensity and were recorded as positive strains. The green fluorescence of O1, one of the positive strains, in 365 nm ultraviolet light was showed in Figure 1a, but there was no fluorescence observed in the negative strain (Figure 1b).

### 2.2. Confirmation of Positive Strains by HPLC-FLD

The OTA-producing ability of the above positive strains was confirmed by HPLC-FLD. The HPLC results confirmed that five of the seven positive strains could produce OTA (Table 1). Therefore, two false positive strains analyzed by HPLC-FLD were screened out and the isolates O1, Q3, V5-1, MQ-5 and MB1-1 have been confirmed as OTA-producing strains. 

The OTA produced by the toxicogenic strains cultured at 25 °C for seven (*Aspergillus* isolate) or 14 days (other isolates) was respectively higher than that produced by corresponding strains cultured in other conditions (data not shown). The maximum and minimum concentrations of OTA production were 97.5 ng/g by O1 and 25.9 ng/g by MB1-1, respectively. The OTA produced by the five strains (Table 1) were all significantly higher (*p* < 0.05) than the minimum level determined by the HPLC-FLD method established for OTA production in grapes.

### 2.3. Identification of OTA-Producing Fungi

**Figure 2:**
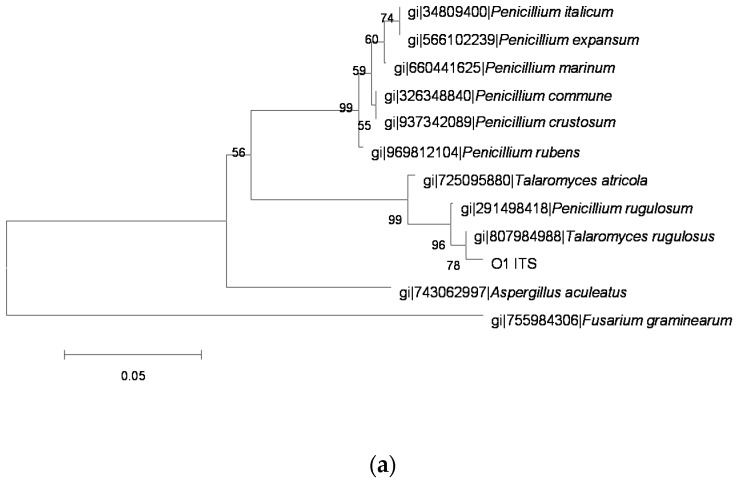

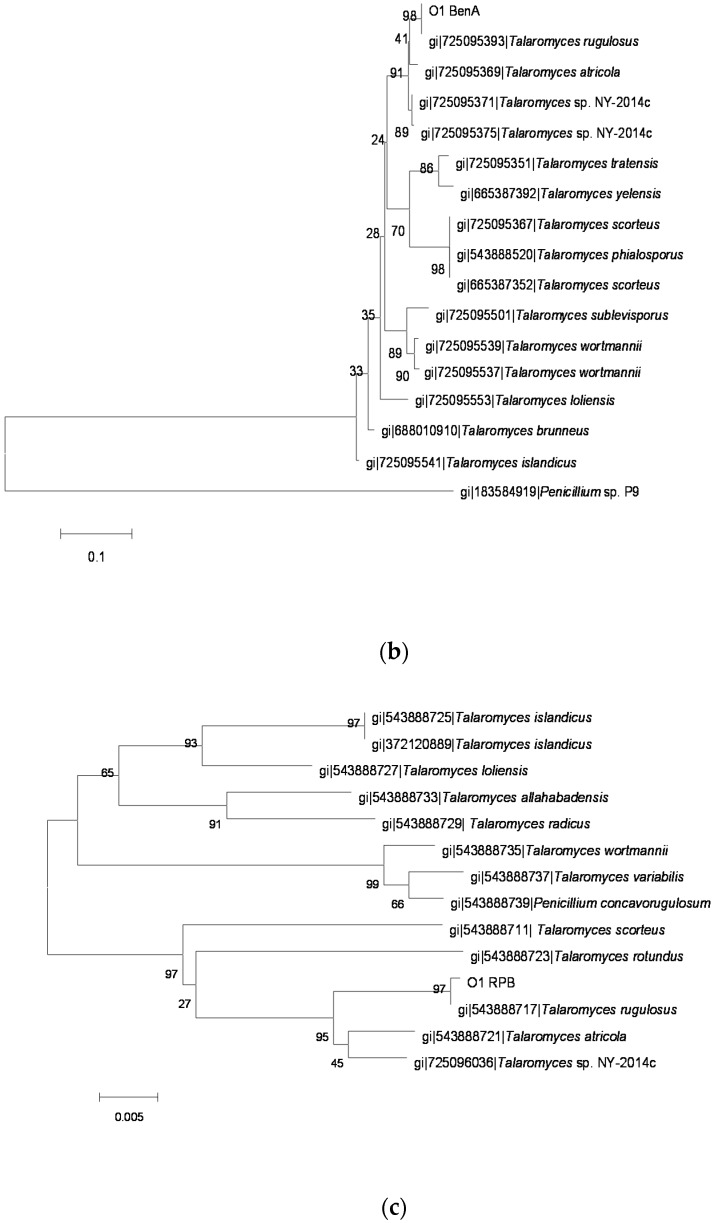
The phylogenetic tree of the O1 strain by the Neighbor-Joining method based on analysis of *ITS*, *BenA* and *RPB2* sequences. (**a**) the phylogenetic tree based on analysis of *ITS*; (**b**) the phylogenetic tree based on analysis of *BenA*; and (**c**) the phylogenetic tree based on analysis of *RPB2*.

**Figure 3:**
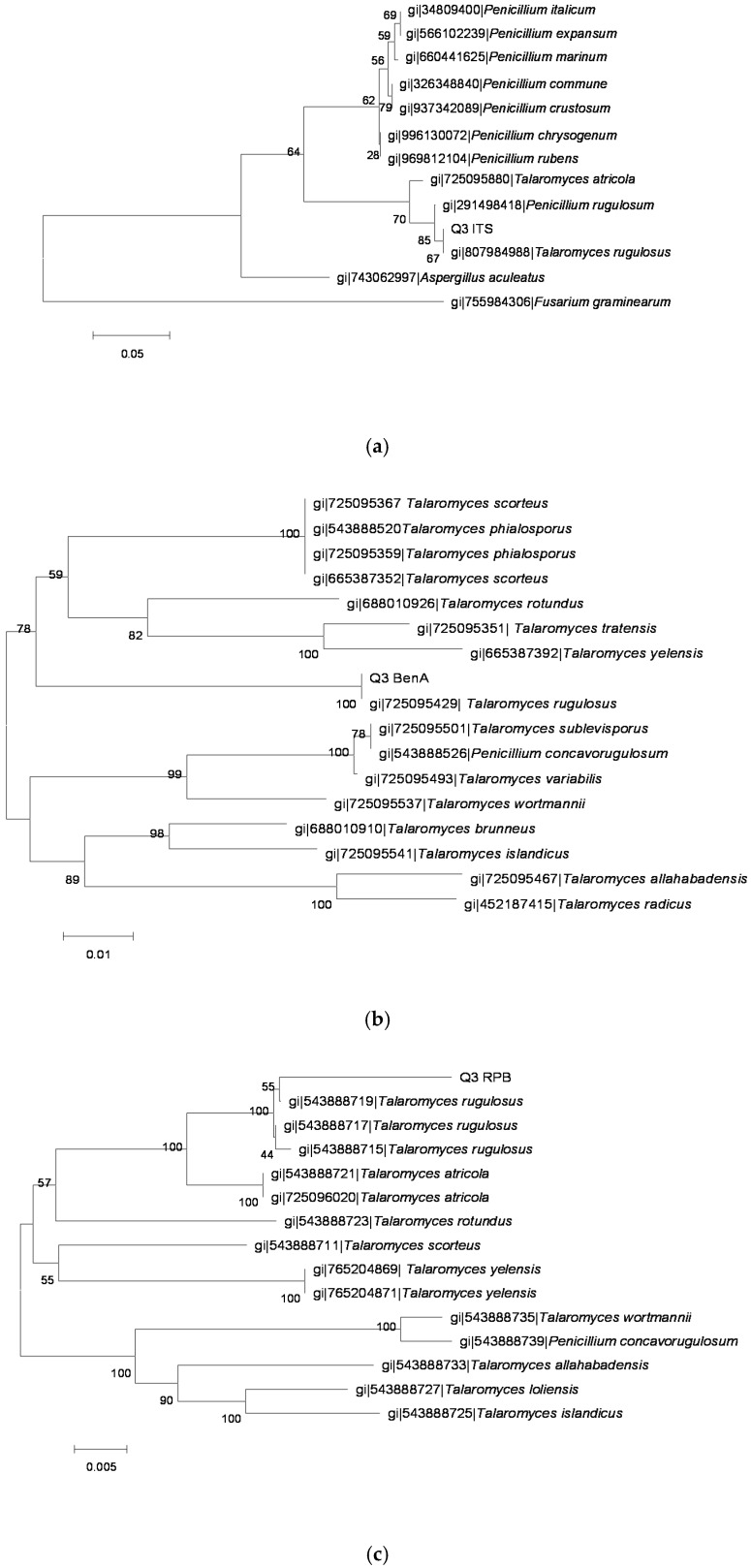
The phylogenetic tree of the Q3 strain by the Neighbor-Joining method based on analysis of *ITS*, *BenA* and *RPB2* sequences. (**a**) the phylogenetic tree based on analysis of *ITS*; (**b**) the phylogenetic tree based on analysis of *BenA*; and (**c**) the phylogenetic tree based on analysis of *RPB2*.

**Figure 4:**
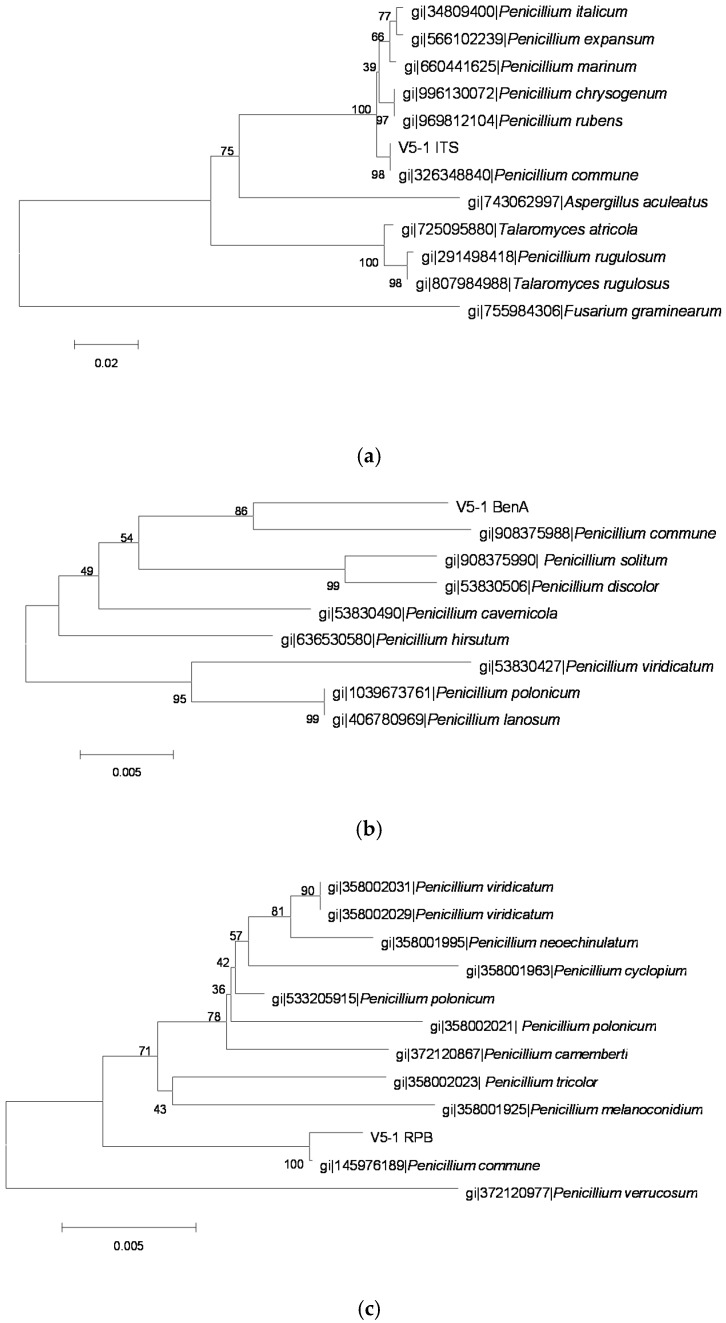
The phylogenetic tree of the V5-1 strain by the Neighbor-Joining method based on analysis of *ITS*, *BenA.* and *RPB2* sequences. (**a**) The phylogenetic tree based on analysis of *ITS*; (**b**) the phylogenetic tree based on analysis of *BenA*; and (**c**) the phylogenetic tree based on analysis of *RPB2*.

**Figure 5:**
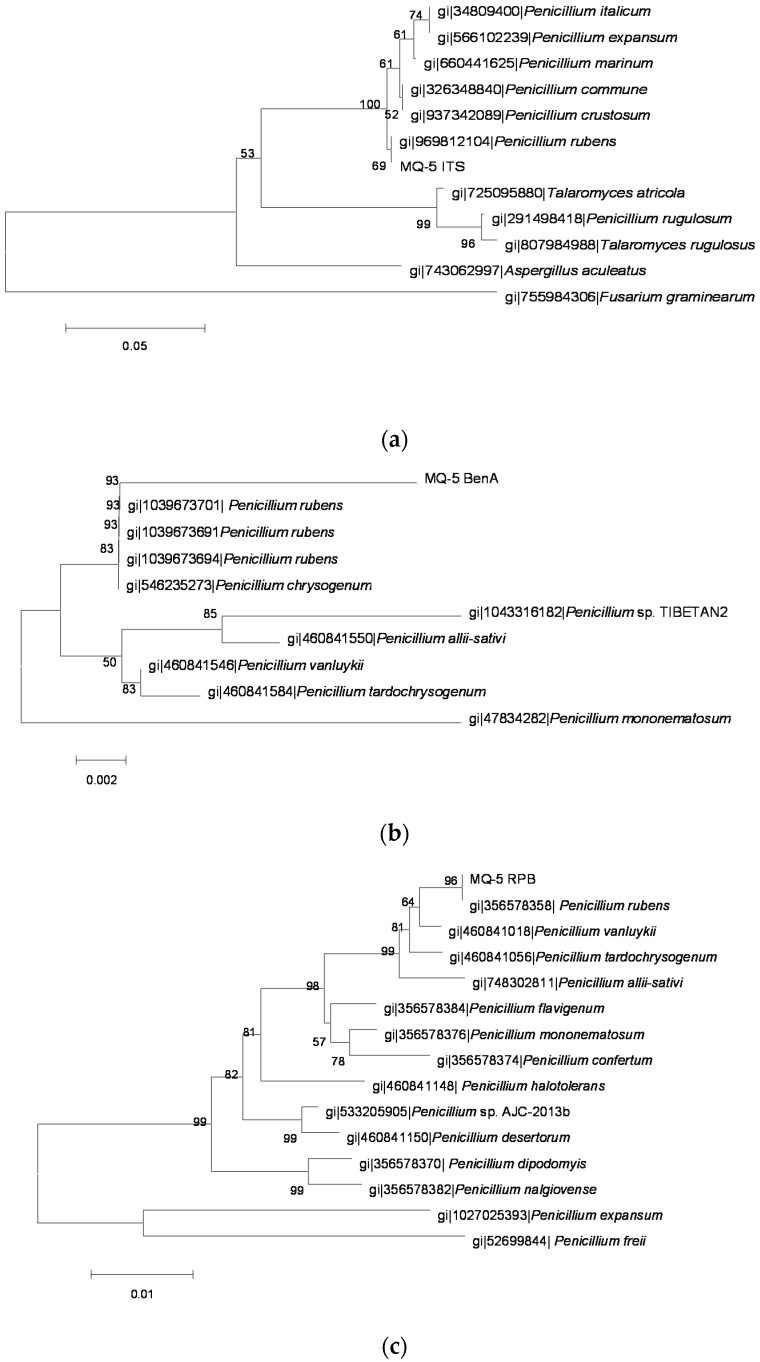
The phylogenetic tree of the MQ5 strain by the Neighbor-Joining method based on analysis of *ITS*, *BenA* and *RPB2* sequences. (**a**) the phylogenetic tree based on analysis of *ITS*; (**b**) the phylogenetic tree based on analysis of *BenA*; and (**c**) the phylogenetic tree based on analysis of *RPB2*.

**Figure 6:**
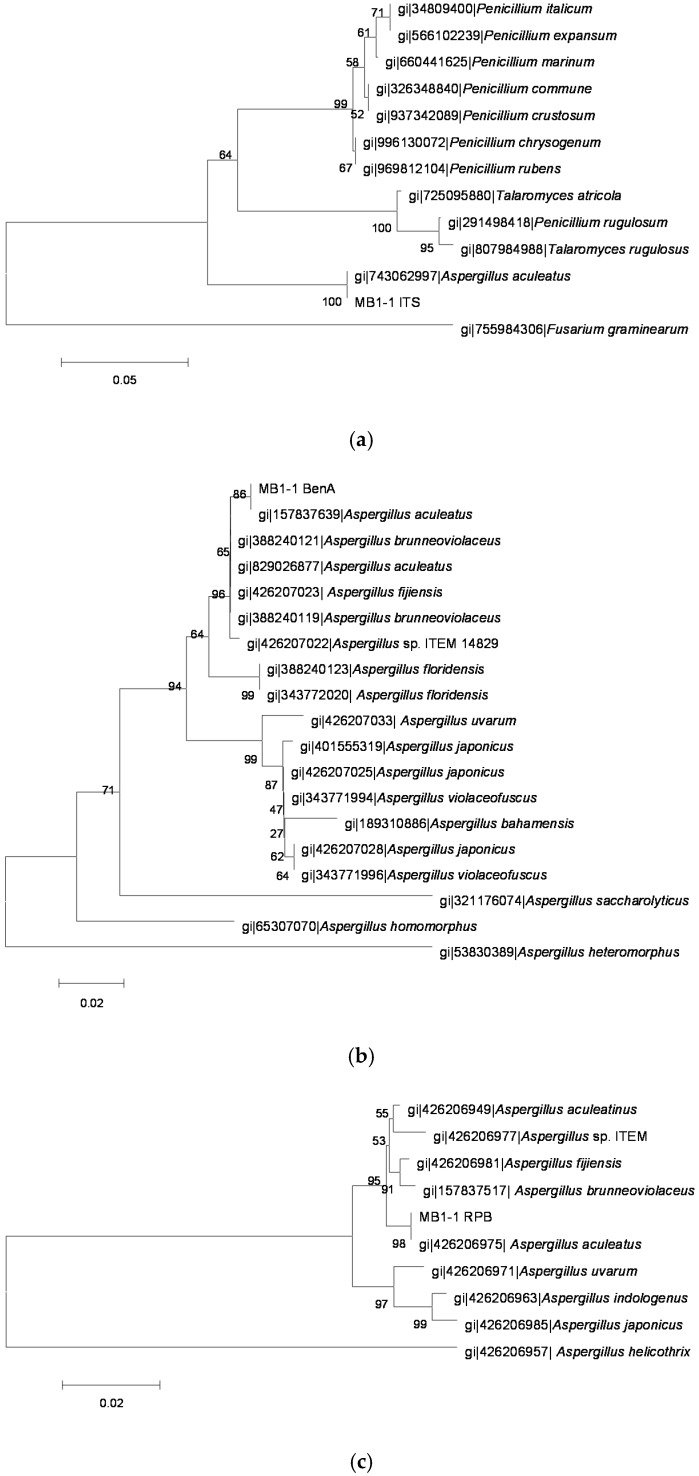
The phylogenetic tree of the MB1-1 strain by the Neighbor-Joining method based on analysis of *ITS*, *BenA* and *RPB2* sequences. (**a**) The phylogenetic tree based on analysis of *ITS*; (**b**) the phylogenetic tree based on analysis of *BenA*; and (**c**) the phylogenetic tree based on analysis of *RPB2*.

The sequences of PCR amplification products were analyzed and molecular phylogenetic trees (Figure 2, Figure 3, Figure 4, Figure 5 and Figure 6) were constructed based on comparative sequence analysis of *ITS*, *BenA* and *RPB2* of the five strains. As shown in Figure 2 and Figure 3, the *ITS*, *BenA* and *RPB2* of the strain O1 and Q3 have high homology with *Talaromyces rugulosus*. The strain V5-1 is *Penicillium commune* because of the *ITS*, *BenA* and *RPB* are all homology with *Penicillium commune* (Figure 4). Similarly, the MQ-5 is *Penicillium rubens* (Figure 5) and MB1-1 is *Aspergillus aculeatus* (Figure 6)*.*

## 3. Discussion

Of the five toxicogenic strains, there are two *Penicillium* species (V5-1 and MQ-5), two *Talaromyces* species (O1 and Q3) and one *Aspergillus* species (MB1-1). In tropical regions of the world, *Aspergillus* species, such as *A. ochraceus* and *A. carbonarius*, are generally the OTA-producing species. However, in temperate regions, OTA is produced by *Penicillium* species, mainly by *P. verrucosum* [20,33]. Zhenjiang is situated in the region where there is a transitional climate from temperate to subtropical climate. Among the five strains isolated from the grapes in the vineyards of Zhenjiang, *Penicillium* species and *Talaromyces rugulosus* (synonyms or basionym of *Penicillium tardum*, *Penicillium chrysitis* and *Penicillium rugulosum*) were the dominant OTA-producing strains. This result is in accordance with the distribution characteristics of OTA-producing strains in different climates reported by Stander et al [33].

*P. Commune*, one of the five OTA-producing strains, is the most frequently occurring spoilage fungus on cheese [34] and some other foods. Many years ago, it was reported that this mold could produce mycotoxins such as OTA [35] and cyclopiazonic acid [36] in contaminated foods.

*P. rubens* (formerly known as *Penicillium chrysogenum*) is frequently found in indoor environments and foods. It has been confirmed as Fleming’s original penicillin producing strain [37] and has gained much attention for its ability to produce penicillin, but there is no report about OTA production. For the first time in this study, it was found that *P. rubens* has the ability to produce OTA.

As Peterson et al [38] reported, *P. tardum* and *P. chrysitis* were synonyms of *T. rugulosus* and *P. rugulosum* is basionym of *T. rugulosus*. The reports about the four strains are very few. Tatsuno et al. [39] confirmed that the cytotoxic metabolic substances of *P. tardum* were endocrocin and emodin, and the known toxic metabolites, rugulosin and skyrin were also isolated. However, the studies about OTA produced by the four strains mentioned above have not been reported so far.

*A. aculeatus* is one member of the Trichocomaceae family [40] and usually found in foods. Two toxic metabolites previously isolated from *Penicillium oralicum*, secalonic acid D and F, were separated from the extrolite profiles of *A. aculeatus* [41,42]. The OTA production ability of 189 strains of black aspergilli on grapes from Europe and Israel were studied by DNA-based molecular methods. The results showed that *A. aculeatus* was unable to produce OTA [43]. It was also found that none of the isolates belonging to *A. niger* aggregate and *A. japonicus var. aculeatus* (obligative or homotypic synonyms format of *A. aculeatus*) from Spanish wine grapes were able to produce OTA [44]. 

In conclusion, O1 and Q3 (*Talaromyces rugulosus*), V5-1 (*Penicillium commune*)*,* MQ-5 (*Penicillium rubens*) and MB1-1 (*Aspergillus aculeatus* ) were OTA-producing molds from grapes before harvest. Up to the period of this study, there has not been any reports on OTA produced by *T. rugulosus* (or *P. tardum*, *P. chrysitis*, *P. rugulosus*), *P. Rubens* and *A. aculeatus*. This is the first study to report that *T. rugulosus*, *P. rubens* and *A. aculeatus* strains with relatively strong abilities of OTA production were isolated from grapes.

Furthermore, these results showed that OTA production is a latent risk if the grapes contaminated with these OTA-producing species are not handled properly. Therefore, it is essential to take measures to avoid contamination by fungi and OTA production in grapes in both growth and post-harvest stages; for example, avoiding exposition to high temperature, minimizing grape accumulation in high relative humidity during harvest and wine making processes and biological controls with antagonistic yeast [45]. The initial discovery of the three OTA-producing strains (*P. rubens*, *T. rugulosus* and *A. Aculeatus*) in grapes is very important. Consequently, this work would contribute to the in-depth understanding of the fungi with OTA-producing ability in grapes. Lastly, it is necessary for researchers to broaden the scope of study and take effective measures to ensure the safety of grapes and its products.

## 4. Conclusions

In conclusion, five molds were screened out from grapes before harvest and identified as *Talaromyces rugulosus* (O1 and Q3), *Penicillium commune* (V5-1), *Penicillium rubens* (MQ-5) and *Aspergillus aculeatus* (MB1-1). This is the first report that *T. rugulosus*, *P. rubens* and *A. aculeatus* strains isolated from grapes can produce OTA.

## 5. Materials and Methods

### 5.1. Samples

Fifteen bunches of grapes infected with diseases (downy mildew, gray mold and white rot) were collected randomly from each one of 3 organic vineyards in Zhenjiang, China. The samples were immediately put in sterilized paper bags and stored at 4 °C, and then were transferred to the laboratory where they were processed.

### 5.2. Culture Medium

Potato Dextrose Agar media (PDA, g/L): potato 200, glucose 20 and agar 20.

Czapek’s Agar medium (CA, g/L): sucrose 30, NaNO_3_ 3, KCl 0.5, MgSO_4_∙7H_2_O 0.5, FeSO_4_·7H_2_O 0.01, K_2_HPO_4_ 1, agar 20.

Coconut Cream Agar medium (CCA, g/L): coconut milk 50%, distilled water 50%, agar 20 g/L.

Rose Bengal medium (g/L): peptone 5, glucose 10, KH2PO4 1, MgSO4∙7H2O 0.5, Rose Bengal 0.0333, chloramphenicol 0.1, agar 20.

All the culture media were prepared with distilled water and were autoclaved at 121 °C for 20 min.

### 5.3. Isolation of Fungi from Decay Grapes

The disease positions of moldy berries were plated onto Rose Bengal medium in Petri dishes by sterilized tweezers. All plates were incubated at 25 °C until colonies were formed followed by repeatedly streaking inoculation of each colony on Rose Bengal medium until single mold colonies were obtained. The three-point inoculation method was employed to foster the purification of fungal strains, which were used for the subsequent experiment. 

### 5.4. Preliminary Screening of Potential OTA-Producing Isolates by Ultraviolet Light

*Aspergillus* isolates were incubated on CCA at 25 °C for 7 days, and other isolates were cultured on CA at 25 °C for 14 days. Then, the reverse of CCA or CA with colonies was exposed to long wavelength UV light (365 nm) in the dark environment [8,46,47]. OTA-producing isolates would take on characteristic green fluorescence. Isolates producing fluorescence were recorded as potential OTA-producing strains. Then, the suspected strains were fumigated with 25%–28% ammonia in dark. The strains that showed enhanced fluorescence intensity under the 365 nm UV light were recorded as positive strains.

### 5.5. Confirmation of OTA-Producing Strains by High-Pressure Liquid Chromatography with a Fluorescence Detector (HPLC-FLD)

OTA produced by the positive strains was extracted by a variation of a method described by Cabañes et al. [48]. All the strains were cultured as above. Three agar plugs (the diameter was 6 mm) were removed from the inner, middle and outer area of each colony of screened positive strains and extracted with 500 μL methanol for 1 hour in darkness. The extracts were vortexed and filtered with 0.22 um filter and frozen (−18 °C) until analysis by HPLC-FLD. 

OTA production was detected with an Agilent Technologies 1100 (Agilent, Santa Clara, CA, USA) series liquid chromatographic system equipped with a scanning fluorescence detector (λexc 330 nm; λem 460 nm). A reversed phase analytical column (Agilent ZORBAX SB -C18, 5 μm, 2 4.6 × 250 mm, Agilent, Santa Clara, CA, USA) was used with a mobile phase of acetonitrile (HPLC grade)—1% acetic acid (60:40, *v*/*v*) at a flow rate of 1 mL/min. The volume of injection was 20 μL and detection temperature was 30 °C [49,50]. The detection limit of the OTA analysis was 2 ng/mL, based on a signal-to-noise ratio of 3:1.

The OTA standard (Pribolab Pte. Ltd, Singapore) solution was prepared in methanol (HPLC grade) and confirmed by HPLC-FLD as above. The calibration curve (25 ng/mL to 2 µg/mL) was drawn for quantifying the OTA in extracts. 

### 5.6. Identification of OTA-Producing Fungi

#### 5.6.1. Genomic DNA Extraction

Fungal strains were cultured in PDB at 25 °C and 180 rpm for 4 days before DNA extraction. The mycelia were collected and freeze-dried, and then ground to powder with liquid nitrogen. The mycelia powder (200 mg) added with 700 μL of lysis buffer (2% CTAB, 1.4 M NaCl, 20 mM ethylene diamine tetraacetic acid (EDTA), 100 mM Tris-HCl pH 8) were incubated at 65 °C for 1 h and then cooled on ice for 1 h. The subsequent procedures of genomic DNA extraction were in accordance with the method described by Tannous et al [51]. The DNA concentration was measured using a Nanodrop2000 Spectrophotometer (Thermo Scientific, Waltham, MA, USA).

#### 5.6.2. PCR Amplification and Sequencing

The conserved genes used to identify the fungi were *ITS*, beta-tublin gene (*BenA*) and RNA polymerase II second largest subunit (*RPB2*) [52,53,54,55]. The primers of the three genes were as follows. *ITS*: ITS1:GGTGAACCTGCGG; ITS4: TCCTCCGCTTATTGATATGC. *BenA*: Bt2a (Forward) GGTAACCAAATCGGTGCTGCTTTC; Bt2b (Reverse) ACCCTCAGTGTAGTGACCCTTGGC. *RPB2*: 5F-Eur (Forward): GAYGAYCGKGAYCAYTTCGG; 7CR-Eur (Reverse): CCCATRGCYTGYTTRCCCAT. The PCR reaction (25 µL) mixture contained 2 μL DNA template, 2.5 μL PCR buffer (Mg^2+^ plus), 2.5 μL (2.5 μm) dNTP, 2.5 μL primer, 0.2 μL Taq DNA Polymerase and H_2_O up to 25 μL. The amplification protocol is showed in Table 2.

All of the PCR amplification products were analyzed by gel electrophoresis and then sent to Shanghai Sangon Biological Engineering Technology and Services Co., Ltd, China for sequencing. The sequences were blast on National Center for Biotechnology Information (NCBI). Part of the genes homologous with *ITS*, *BenA* and *RPB* was acquired. These homologous genes were clustered by the clustalW software 2.0 (University college Dublin, Dublin, Ireland), and phylogenetic trees based on these sequences were constructed with the neighbor-joining algorithm by MEGA5.0 (University college Dublin, Dublin, Ireland).

## Figures and Tables

**Table 1:** Ochratoxin A (OTA) concentration produced by the five positive strains analyzed by high-pressure liquid chromatography with a fluorescence detector (HPLC-FLD). Each value is the mean of three experiments.

Isolates	OTA Content (ng/g)
O1	97.5 ± 2.8
Q3	50.11 ± 2.1
V5-1	43.94 ± 1.8
MQ-5	96.86 ± 3.2
MB1-1	25.9 ± 1.9

**Table 2:** PCR amplification protocol.

Gene	Profile Type	Initial Denaturing	Cycles	Denaturing Annealing Elongation	Final Elongation	Rest Period
*ITS*, *BenA*	standard	94 °C, 10 min	35	94 °C, 45 s; 55 °C, 45 s; 72 °C, 60 s	72 °C, 10 min	4 °C, ∞
*RPB*	touch-up	94 °C, 5 min	5	94 °C, 45 s; 50 °C, 45 s; 72 °C, 60 s	-	-
-	-	-	5	94 °C, 45 s; 52 °C, 45 s; 72 °C, 60 s	-	-
-	-	-	30	94 °C, 45 s; 55 °C, 45 s; 72 °C, 60 s	72 °C, 10 min	4 °C, ∞
